# Delayed emergence of behavioral and electrophysiological effects following juvenile ketamine exposure in mice

**DOI:** 10.1038/tp.2015.111

**Published:** 2015-09-15

**Authors:** L R Nagy, R E Featherstone, C G Hahn, S J Siegel

**Affiliations:** 1 Department of Psychiatry, Translational Neuroscience Program, University of Pennsylvania, Philadelphia, PA, USA

## Abstract

Frequent ketamine abuse in adulthood correlates with increased risk of psychosis, as well as cognitive deficits, including disruption of higher-order executive function and memory formation. Although the primary abusers of ketamine are adolescents and young adults, few studies have evaluated its effects on juvenile cognition. Therefore, the current study analyzes the effect of adolescent ketamine exposure on cognitive development. Juvenile mice (4 weeks of age) were exposed to chronic ketamine (20 mg kg^−1^, i.p. daily) for 14 days. Mice were tested immediately after exposure in the juvenile period (7 weeks of age) and again as adults (12 weeks of age). Measures included electroencephalography (EEG) in response to auditory stimulation, the social choice test, and a 6-arm radial water maze task. Outcome measures include low-frequency EEG responses, event-related potential (ERP) amplitudes, indices of social behavior and indices of spatial working memory. Juvenile exposure to ketamine was associated with electrophysiological abnormalities in adulthood, particularly in induced theta power and the P80 ERP. The social choice test revealed that ketamine-exposed mice failed to exhibit the same age-related decrease in social interaction time as controls. Ketamine-exposed mice outperformed control mice as juveniles on the radial water maze task, but did not show the same age-related improvement as adult controls. These data support the hypothesis that juvenile exposure to ketamine produces long-lasting changes in brain function that are characterized by a failure to progress along normal developmental trajectories.

## Introduction

Ketamine is an N-methyl-D-aspartate (NMDA) receptor antagonist used medically for its dissociative anesthetic effects.^1^ The drug is widely abused around the world as a hallucinogen and date rape drug.^1^ Studies have shown widespread ketamine use in city club scenes, particularly in the US, Europe and Asia.^2, 3^ The World Health Organization (WHO) has collected data from 64 countries around the world, 25 of which reported harmful ketamine abuse among its youth population.^4^ Ketamine is the most commonly abused drug among Hong Kong youth,^5^ and some report a large increase in popularity among urban European youth, notably in London.^2^ In the US, trafficking of the drug is known to take place across the Mexican border and from theft from clinics or other legitimate sources.^1^ Sale to adolescents typically takes place inside clubs or at parties.^1^ Recently, physicians have used ketamine for treating patients with severe depression that is unresponsive to common treatment.^6, 7^ Unlike the typical medical usage of ketamine, which generally involves a one-time anesthetic dose, treatment for depression requires multiple low doses of the drug.^6, 7^ The relative success of this treatment and its growing popularity only emphasize the importance of understanding the long-term drug effects on patients of all ages.

In addition to the aforementioned clinical indications, NMDA receptor antagonists such as ketamine are frequently used as neurodevelopmental models for schizophrenia.^8, 9, 10, 11^ It is theorized that schizophrenia patients suffer a loss of NMDA receptor-mediated glutamate signal transduction, which may be effectively simulated by NMDAR antagonists such as ketamine.^8^ Studies have suggested that the effects of ketamine may be similar to symptoms of the disease in humans.^11^ Specifically, psychosis due to chronic ketamine exposure has been shown to exhibit similarities to schizophrenia psychosis in humans.^9^ In addition, ketamine exposure results in functional changes in rodents that are similar to those seen in patients.^8, 12, 13, 14^

Frequent ketamine abuse in adulthood is known to correlate with high risk of psychosis, as well as cognitive deficits, indicating a disruption of higher-order executive function and memory formation.^15^ In human studies, ketamine users were consistently outperformed by controls on both intelligence and memory tasks.^5^ Increases in ketamine use correlate with decreases in spatial working memory function and pattern recognition memory in humans.^16^ Rats and mice administered ketamine as adults have shown signs of permanent cognitive impairment, such as impaired spatial memory^13, 17^ and distinct changes in electroencephalographic (EEG) measures.^13, 18^ In a study following several human drug user groups, ketamine users stood out as showing more perceptual disturbances and a greater likelihood of depression symptoms, as well as decreased verbal fluency, greater attention deficits and difficulty in working memory manipulations when compared with controls and other drug users.^19^

Although the primary abusers of ketamine are adolescents and young adults, few studies have been done to evaluate its effects on juvenile cognitive development. Studies suggest that drug abuse in the adolescent period leaves users with increased vulnerability to cognitive performance deficits, psychosis and neuroanatomical changes that can affect the rest of their lives.^20, 21^ Ketamine administered acutely or chronically in the neonatal period has been shown to cause cognitive deficits, particularly in learning and memory, which carry into adulthood.^22, 23, 24^ Other studies have suggested a heightened sensitivity to the effects of ketamine in the postnatal period.^25, 26, 27, 28^ Ketamine administered to mice in adolescence has suggested the potential for delayed emergence of cognitive impairment through EEG measures, with signs of deficits only appearing in adulthood—similar to schizophrenia, where symptoms such as psychosis typically do not occur until late adolescence or early adulthood.^12^ However, ketamine is currently used as an anesthetic in children, but not in adults, based on a decreased incidence of psychiatric manifestations when the drug is used chronically below the age of 15.^29, 30, 31, 32, 33^

Our study analyzes the effect of adolescent ketamine exposure on cognitive development in mice. Following the chronic exposure of adolescent mice to ketamine or saline vehicle, subjects underwent two stages of assessment, including 1 week following cessation of exposure as juveniles, and again at 5 weeks following cessation as adults. Spatial memory was assessed using a radial arm water maze, social preference was assessed using a social interaction task and EEG indices of brain function were also evaluated, including event-related potential (ERP) and baseline- and event-related power. The radial arm water maze task requires mice to utilize spatial memory to find a platform based on cues in the testing room, and has been used successfully with rats and mice.^34, 35^ Social interaction testing, specifically the social choice or social approach test,^36, 37, 38^ allows controlled measurement of social interactions initiated by the test mouse, and is a replicable measure of sociability in mice.^38^ EEG measures are known to reflect neurodevelopmental changes during the adolescent period,^39, 40, 41^ and changes following ketamine treatment in adult mice are well-characterized.^13, 42, 43^ Furthermore, EEG phenomena, which are in part regulated by the GABA and glutamate systems, are altered in disorders due to abnormal NMDA receptor-mediated glutamate signaling, including ERPs and ER spectral perturbation (ERSP) theta frequencies.^44, 45, 46^ Alterations in power within the theta frequency range following auditory stimuli reflect performance on cognitive tasks including attention, working and long-term memory.^47, 48, 49, 50, 51^ There is also evidence that modulations in theta oscillations are reflective of hippocampal spatial memory function.^48^ As attention and working spatial memory are important aspects of our behavioral testing, theta frequencies are used in this study to shed light on ketamine-induced changes across the lifespan. Furthermore, in a related study, it was discovered that theta power was selectively altered in adult animals following chronic ketamine exposure ^13^

The current study tests the hypotheses that exposure to ketamine during adolescence will cause:

- Delayed emergence of abnormalities in social behavior.
- Delayed emergence of abnormalities in working memory.
- Delayed onset of abnormal patterns of EEG.
- Delayed onset of abnormalities in social, cognitive and EEG measures will reflect a failure to progress rather than a deterioration.

## Materials and methods

### Mice

Juvenile C57/Bl6J male mice from Jackson Laboratories (Bar Harbor, ME, USA) were used. Mice arrived at 21 days of age to our facility and were allowed 1 week for acclimation prior to the start of injections. Mice received 14 days of injections starting at 28 days of age. Electrodes were implanted at 42 days of age, with a week-long recovery period before testing. Mice were tested for all measures at 7–8 weeks of age and again at 11–12 weeks of age. Testing included 1 day of EEG recording, at 49 days and 77 days of age; 1 day of social interaction testing at 50 days and 78 days of age; and 3 days of water maze testing beginning at 51 and 79 days of age. Mice were kept on a 12:12 h light/dark cycle, with chow and water available *ad libitum* over the duration of the study. On conclusion of the last day of testing, mice were killed and brain samples were removed for future studies. All procedures adhered to the guideline of the NIH Guide for the Care and Use of Laboratory Animals and the local Institutional Laboratory Animal Care and Use Committee.

### Injections

Mice received either 0.9% saline (*n*=14) or 20 mg kg^−1^ i.p. ketamine (*n*=14), injected daily over a 14-day period. This dose was chosen based on previous studies^12, 52, 53^

### Electrode implantation

Electrode implantation took place under 1% isoflurane anesthesia. A series of three small holes were drilled into the skull at −1.8, −0.8 and +0.2 mm Anterior-Posterior to bregma; and 2.65 mm lateral. A positive electrode was placed into right hippocampal region at 1.8 mm posterior, 2.65 mm lateral and 2.75 mm dorsal to bregma. A reference electrode was lowered onto the surface of the ipsilateral cortex at 0.2 mm anterior, 2.75 mm lateral and 0.75 mm dorsal.^12, 13, 46, 54^ A ground electrode was lowered onto the cortical surface between the positive and reference electrodes at 0.8 mm posterior, 2.75 mm lateral and 0.75 mm dorsal. Ethyl cyanoacrylate (Loctite, Henkel, Duesseldorf, Germany)^13^ and dental cement (Ortho Jet, Lang Dental, Wheeling, IL, USA) were used to secure the electrodes to the skull.^55, 56^ One week of rest was given to allow for recovery before EEG recording took place.

### EEG recording and analysis

The EEG testing apparatus consisted of eight standard mouse cages, modified to allow placement of a speaker on the top of the cage for delivery of auditory stimuli, located within a Faraday cage. Auditory stimuli were generated by Micro1401 hardware and Spike2, version 6.0 (Cambridge Electronic Design, Cambridge, UK). EEG was recorded during auditory click presentations (8-s inter-stimulus interval) of a white noise tone (10 ms duration, 85 dB). Mice received a total of 300 clicks. To minimize the effects of stress, animals were given 15 min to acclimate to the ERP apparatus prior to each ERP recording session. ERPs were analyzed using Spike2 software.

### EEG measures

EEG data were processed using EEGLAB. Several mice were excluded from the EEG testing due to poor signals or broken electrodes.

### Event-related potentials

ERP is often used to translate sensory and cognitive changes in schizophrenia models to the patient.^57^ ERPs are systematic voltage deflections in the averages of EEG recorded during a response to repeated events, as described above; as average value of EEG activity during a repeated stimulus, ERP is believed to reflect bursts of neural activity that are ‘time locked' to these stimulus events.^57^ The amplitude of ERP waveform components were quantified as the maximum or minimum amplitude changes relative to the previous points of inflection. Latency was calculated as the time point at which the maximum or minimum value occurred during the specified time interval. P20 amplitude was calculated as the maximum value between 10 and 30 ms; N40 amplitude is defined as the minimum value between 25 and 60 ms; and P80 amplitude is defined as the maximum value between 50 and 200 ms.

### Event-related spectral perturbation

ERSP is a reflection of the degree of power at a particular frequency range; in this case, we explored the ERSP data for theta oscillations. Theta frequency ERSP was quantified as the average EEG power between 4 and 12 Hz between 0 and 200 ms post stimulus. Power reflects the oscillation amplitude.^54^ Baseline measurements were gathered from data prior to the start of auditory stimuli, when raw EEG was recorded for a 60-s period. Inter-trial coherence (ITC) measures the degree of cross-trial entrainment of the oscillation at a particular time-point for a particular frequency range. ITC is calculated by measuring the phase angle of the oscillation, with a value of 1 being complete overlap and 0 being no overlap between cross-trials. ITC was measured in the theta frequency range.

### Radial arm water maze testing and analysis

**Figure 1:**
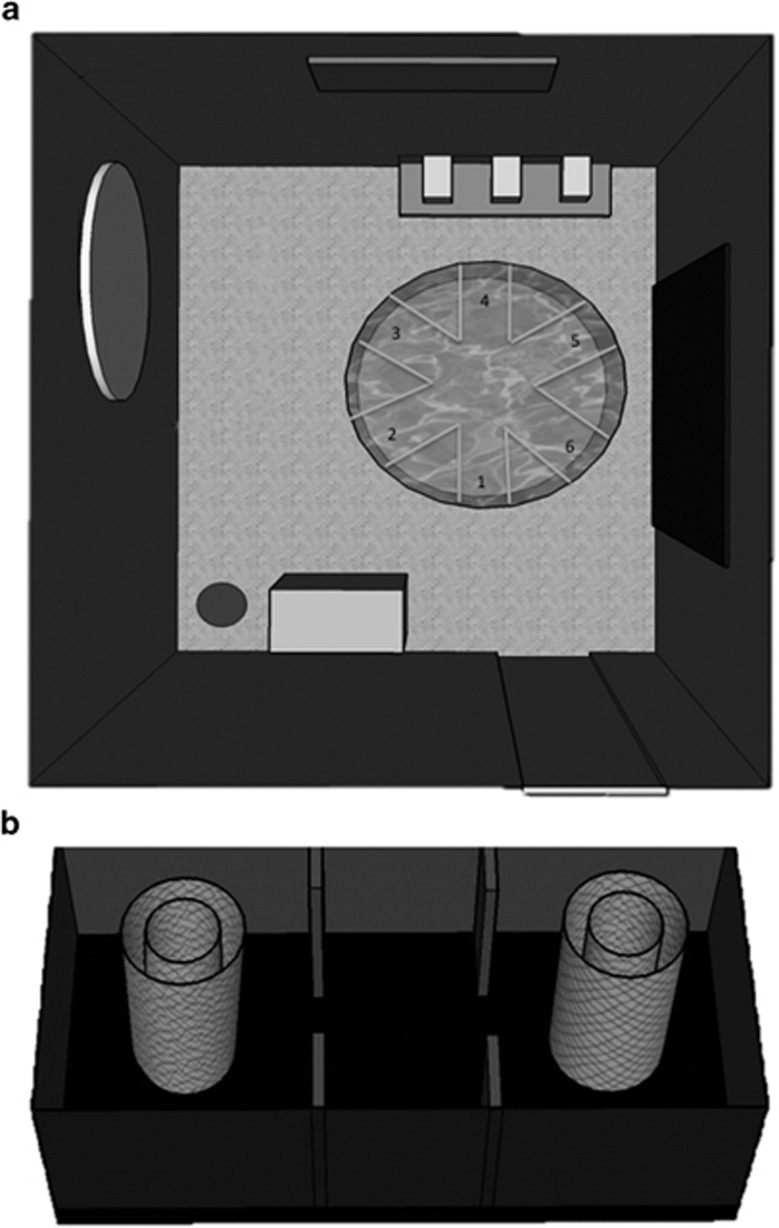
Schematic representation of behavioral testing arenas. (**a**) Radial arm water maze. The layout of the maze with visual cues on the walls is indicated. The dark gray circle indicates the spot at which the test administrator stands in relationship to the room. (**b**) Social approach test 3-chambered arena. During the testing period a live gonadectomized peer mouse is placed in one cylinder, while an inanimate object of similar size and shape is placed in other. Video recordings were made with an overhead view to analyze the social interaction data.

The radial arm water maze consists of a circular pool, 90- cm wide by 78-cm high, with six stainless-steel arms of length 25.4 cm and width 20 cm, creating wide paths and an open center area. The pool was filled to 65 cm, submerging a platform placed at the end of one arm by about 3 cm of water. Non-toxic white paint was used to hide any distinguishing features of the maze beneath the waterline, including the platform. Lights were dimmed, and numerous visual cues were placed on walls throughout the room to act as visual cues to help the mice to navigate the maze (Figure 1a).

Testing was performed for 3 days, with 5 trials per day. At the start of each trial, mice were placed in one of the five empty arms. Start locations were randomized so that no arm was repeated, no mouse started from adjacent arms in consecutive trials and no sequence of arms was repeated. Each day, every mouse swam a full course of five arms, and was allowed 60 s to find the platform on each trial. If mice failed to find the platform in this time, they were guided to the platform before being removed from the maze. Mice were removed from the platform after 10 s, dried and placed in a warming chamber for 10 min before undergoing subsequent trials with different start locations. For juvenile testing, the platform was always placed in arm 5; for adult testing, the platform was always placed in arm 3, to prevent mice from using any long-term spatial memory to complete the task.

The performance of mice on each trial was recorded by a single researcher who stood in the same place in the room for each trial. Latency and order of entry into maze arms during each trial were measured for every mouse by the same researcher. The number of errors (accuracy) and latency (the time taken to complete the maze) was analyzed. Efficiency was measured by analyzing the time spent per arm entry.

### Social interaction testing and analysis

The social preference of subject mice was tested using the controlled social approach (or social choice) test.^58^ The test is performed in a three chambered box (27 × 53 × 23 cm^3^), as shown in Figure 1b. A perforated cylinder is placed in both end chambers. On test day, subject mice were given a 15-min acclimation period in which no stimuli were placed in the chamber. Subject mice were then removed and the chamber was prepared for the 5-min testing period. A gonadectomized male A/J mouse (Jackson Laboratories), of the same age as the subject mice, was placed inside of one of the cylinders, while an inanimate object the size of a mouse was placed in the opposite cylinder. The size of the cylinder allows the stimulus mouse enough room to turn around, and the perforated holes on the surface are large enough to allow for sniffing or touching social interaction initiated by the subject mouse. The constriction placed on the stimulus mouse allows only the subject mouse to initiate social interaction. Therefore, this paradigm favors evaluating the sociability initiated by the subject mouse. During the 5-min testing period, the subject mouse is placed in the test box and is free to initiate or avoid contact with the stimulus mouse. Testing was recorded by Topscan video software (Version 2, Clever Sys, Reston, VA, USA). Videos were scored using MATLAB social counter software. Data on the number of social interactions, defined by sniffing behavior toward the stimulus mouse, and time spent on social activity, were gathered using this program (Figure 1b).

### Statistical analysis

Statistical analyses were performed using STATISTICA (version 6, StatSoft, Tulsa, OK, USA). For the water maze data, statistical analysis was conducted using a 2 × 3 × 5 way repeated measures analysis of variance with an alpha threshold of *P*=0.05. A two-way repeated measures analysis of variance was used for both the social interaction and the EEG analyses. Fisher least significant difference *post hoc* analyses were used.

## Results

### Electroencephalography

#### Event-related potential

**Figure 2:**
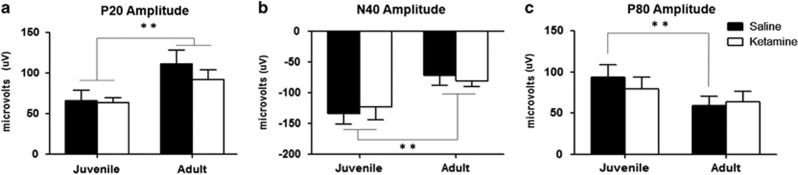
Theta event-related spectral perturbation (ERSP) in ketamine-treated and control mice as juveniles and adults. Ketamine mice are depicted in white and controls in black. (**a**) P20 amplitude. P20 peak amplitude decreases significantly with age, but with no effect of ketamine (F_1,16_=29.133, *P*=0.00006). (**b**) N40 amplitude. A significant decrease in the N40 peak amplitude is observed across the two time-points, with no effect of ketamine (F_1,16_=15.740, *P*=0.00111). (**c**) P80 amplitude. A significant age-related decrease in the P80 peak is observed only in saline-treated mice. (F_1,16_=6.1290, *P*=0.02486, *post hoc*: *P*=0.039409 for juvenile vs adult saline mice). **P<0.05.

P20 amplitude increased with age across both treatment groups (F_1,16_=29.133, *P*=0.00006, Figure 2a). An age-related decrease in the amplitude of the N40 peak was also observed across both treatment groups (F_1,16_=15.740, *P*=0.00111, Figure 2b). An age-related decrease in P80 amplitude was observed, with a *post hoc* revealing an age-related change only in vehicle-treated mice (F_1,16_=6.1290, *P*=0.02486, *post hoc*: *P*=0.039409 for juvenile vs adult saline mice, Figure 2c).

#### Event-related spectral perturbation

**Figure 3:**
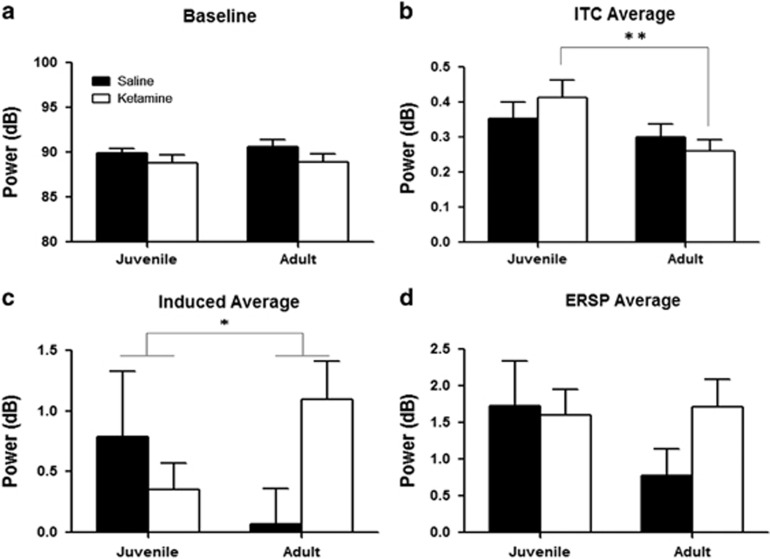
Theta event-related spectral perturbation (ERSP) and time-frequency analysis data in ketamine-treated and control mice as juveniles and adults. Ketamine mice are depicted in white, controls in black. (**a**) Theta baseline power activity in mice as juveniles and adults. No changes in theta baseline activity levels were observed. (**b**): Theta ITC. An effect of age was observed in ketamine-treated mice that was not observed in controls (F_1,16_=0.7958, *P*=0.38, *post hoc*: *P*=0.006 for ketamine-treated juvenile vs ketamine-treated adult, NS in saline-treated mice). (**c**) Theta-induced power activity. As juveniles, both treatment groups showed a similar power. As adults, a reduction of theta power is observed in control mice while an increase is observed in ketamine-treated mice (F_1,16_=6.8882, *P*=0.01840). (**d**) Theta ERSP. Data indicate that there is a trend for a reduction of ERSP with aging in controls and a failure to progress similarly over time in ketamine-treated mice (F_1,16_=4.0956, *P*=0.06003, *post hoc*: *P*<0.05 for saline mice, NS in ketamine mice). ITC, inter-trial coherence; NS, not significant. *P<0.05, **P<0.01.

There was no change in baseline theta power (Figure 3a). Theta ITC decreased with age in the ketamine group but not the saline group (F_1,16_=0.7958, *P*=0.38, *post hoc*: *P*=0.006 for ketamine-treated juvenile vs ketamine-treated adult, not significant (NS) in saline-treated mice, Figure 3b). There was no significant interaction across ages or treatment groups for theta-evoked power. A significant interaction of age and treatment group was observed in the theta-induced power such that as juveniles both treatment groups showed similar outcomes, while as adults power was reduced in saline-treated relative to ketamine-treated mice (F_1,16_=6.8882, *P*=0.01840, Figure 3c). Theta ERSP, a reflection of both evoked and induced theta power, showed a trend toward reduction as adults in saline-treated mice (*post hoc*: *P*<0.05). However, there was no change across development in ketamine-treated mice (*post hoc*: *P*=0.8; F_1,16_=4.0956, *P*=0.06003, interaction of treatment group and ketamine treatment, Figure 3d).

#### Social interaction

**Figure 4:**
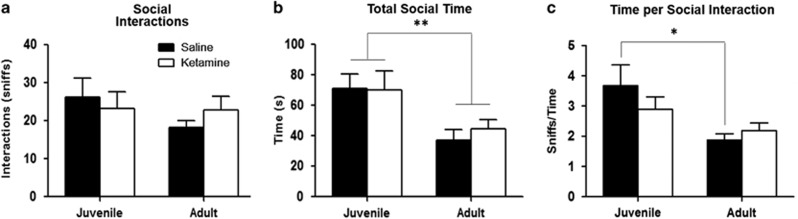
Social approach test comparing juvenile and adult time-points. Ketamine-treated mice are depicted in white, controls in black. (**a**) Number of social interactions during the testing period. No significant difference was observed between the number of social interactions in mice across treatment group or age. (**b**) Total time spent on social interaction during the 5-min testing period. Adult mice showed a significant decrease in overall time spent on social interaction compared with juvenile mice, regardless of drug treatment (F_1,25_=21.842, *P*=0.00009). (**c**) Average time spent per social interaction. Saline mice show a significant decrease in time spent per social interaction as they age, while ketamine mice show no significant change (F_1,25_=8.827, *P*=0.007, *post hoc*: *P*=0.0082 for saline-treated juvenile vs adult mice, NS in ketamine-treated mice). NS, not significant. *P<0.01, **P<0.001.

There were no significant effects of age or drug treatment on the number of social interactions (Figure 4a). A significant age-related decrease in total time spent on social interaction was observed in both groups (F_1,25_=21.842, *P*=0.00009, Figure 4b). *Post hoc* analyses revealed saline-treated animals show an age-related decrease in time spent per social interaction, where ketamine-treated mice did not (F_1,25_=8.827, *P*=0.007, *post hoc*: *P*=0.0082 for saline-treated juvenile vs adult mice, NS in ketamine-treated mice, Figure 4c).

### Water maze

#### Accuracy

**Figure 5:**
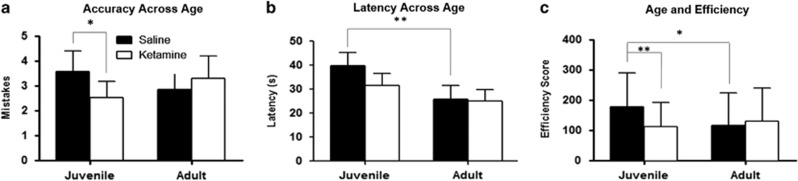
Six arm radial water maze. Ketamine-treated mice are represented in white, and controls in black. (**a**) Accuracy across age by treatment group. Ketamine-treated mice made fewer mistakes as juveniles and decreased their accuracy as adults. Saline-treated mice displayed less accuracy as juveniles and showed fewer mistakes as adults (F_1,23_=5.2019, *P*=0.03215). Both groups showed similar levels of adulthood accuracy. (**b**) Latency measure detailing the time spent on maze completion in each treatment group over two time-points. Latency decreases significantly as control mice age, while no change in ketamine-treated mice was observed (F_1,23_=12.248, *P*=0.00193, *post hoc*: saline juvenile to saline adult *P*=0.002, Ketamine juvenile to ketamine adult *P*=0.124). (**c**) Efficiency scores in both treatment groups with age. As juveniles, ketamine-treated mice show greater efficiency than controls. Efficiency decreases in the former and increases in the latter, showing similar efficiency scores in adulthood (F_1,23_=3.8051, *P*=0.064, *post hoc*: *P*<0.01 for K vs S as juveniles, NS as adults). NS, not significant. *P<0.05, **P<0.001.

There was a significant interaction between age and treatment (F_1,23_=5.2019, *P*=0.03215, Figure 5a). A *post hoc* analysis revealed that ketamine-treated mice made fewer mistakes as juveniles than saline-treated mice (*post hoc*: *P*=0.008), but there was no difference between groups in adulthood. A significant improvement in accuracy occurred across both developmental testing points, on all days and among all treatment groups by trial 3, which remained consistent through trials 4 and 5 (F_4,92_=5.2035, *P*=0.0008, *post hoc*: *P*<0.002 comparing trials 1 and 2 to trials 3–5, NS between trials 1 and 2 and trials 3–5, Supplementary Figure 1A).

#### Latency

There was a significant decrease in latency with every subsequent testing day in both treatment groups and across both testing periods (F_2,46_=24.254, *P*=0.00000, Figure 5b). A significant age-related interaction of latency was also observed, where latency decreased significantly in the adulthood testing, and *post hoc* analysis indicates that there is a significant decrease in average latency from juvenile to adulthood only in saline-treated mice (F_1,23_=12.248, *P*=0.00193, *post hoc*: saline juvenile to saline adult *P*=0.002, ketamine juvenile to ketamine adult *P*=0.124, Supplementary Figure 1B). There was a significant reduction in latency in both groups by trial 2, which was observed across both juvenile- and adult-testing points (F_4,92_=3.9955, *P*=0.00493, Supplementary Figure 1C). Latency decreased significantly across each day during juvenile testing and remained at this optimal latency throughout the subsequent 3 days of testing during adulthood (F_2,46_=0.4534, *P*=0.01706, Supplementary Figure 1D).

#### Efficiency

There was a trend toward an interaction between age and treatment group for efficiency. *Post hoc* analysis indicates that ketamine-treated mice display better performance than saline-treated mice as juveniles but not as adults, (F_1,23_=3.8051, *P*=0.064, *post hoc*: *P*<0.01 for ketamine-treated vs saline-treated juvenile mice, NS as adults, Figure 5c). Ketamine-treated mice remained constant as they reached adulthood, while saline-treated mice significantly improved. There was a significant improvement in efficiency after trial 3 across all days and both ages (F_4,92_=4.1047, *P*=0.00418, *post hoc*: *P*<0.05 for trial 1 and 2 vs trials 3–5, NS between 1 and 2 or 3–5, Supplementary Figure 1E). Similarly, there was a significant improvement in efficiency score after day 1 at both ages (F_2,46_=4.1934, *P*=0.02124, *post hoc*: *P*<0.05 for day 1 vs 2 or 3 and day 2 vs 3, Supplementary Figure 1F).

## Discussion

Juvenile exposure to ketamine was associated with electrophysiological abnormalities in adulthood, particularly in induced theta power and the P80 ERP. No other electrophysiological differences were found between ketamine-treated and control mice. Ketamine-exposed mice failed to exhibit the same age-related decrease in social interaction time as controls. Although ketamine-exposed mice outperformed control mice as juveniles on the radial water maze, they did not show the same age-related improvement as control. These data are consistent with the notion that juvenile exposure to ketamine produces long-lasting changes in brain function that may interfere with progression along normal developmental trajectories.

The current study first tested the hypotheses that exposure to ketamine during adolescence would cause a delayed emergence of abnormalities in social behavior. Data revealed that saline-treated mice showed an age-related decrease in time-per-social interaction that was not evident in ketamine-treated mice. All other measures including number of social interactions and total time spent on social interaction showed significant age-related decreases across both groups. This selective effect of ketamine on the length of individual interactions suggests that ketamine-treated mice may fail to respond to cues related to the termination of social contact. Adult male mice are known to be less social overall when compared with juvenile mice, which may reflect progression to a mature post-pubertal pattern of conspecific male interaction.^59^ Data in the current study indicate that prior exposure to NMDAR antagonists led to persistence of a juvenile pattern of social interaction, suggesting that the overall effect of these agents may be to alter developmental trajectories of sociability. Furthermore, a failure to adapt to social feedback over time is consistent with proposed theories related to developmental alterations in NMDAR function among people with adult-onset social dysfunction (for example, schizophrenia). In human subjects, acute ketamine administration results in significant deficits in understanding facial cues for sadness, as well as a trend toward difficulty in identifying negative emotions.^60^ Similarly, schizophrenia patients show significant deficits in emotion recognition tasks, particularly with emotions such as fear and disgust.^61, 62, 63^ These studies may indicate that our mice exhibit a translationally valid effect of delayed emergence of deficits in understanding of social cues in schizophrenia.

Our study also tested the hypothesis that chronic ketamine administration would result in the delayed emergence of abnormalities in working memory. We observed that control mice showed improvement on the spatial memory task in adulthood with a reduction in latency to find targets and resulting efficiency scores. Ketamine-treated mice did not change their performance over time. However, this observation may be in part related to the finding that ketamine-treated mice performed better than their control counterparts as juveniles. Furthermore, juvenile ketamine mice performed at a level that was similar to a control adult level. Therefore, data do not support the hypothesis that ketamine abuse in adolescence causes delayed emergence of abnormalities in working memory, though it is interesting to note that it does alter the normal trajectory for spatial working memory development. The failure to progress to more successful outcomes in ketamine-treated mice may reflect a ceiling effect, in which mice cannot perform significantly better than the level already achieved by them as juveniles. Previous studies demonstrate that mice typically achieve error rates of no fewer than three mistakes at their most successful, suggesting that our ketamine-treated mice could not have improved from their juvenile rates.^64, 65^ In addition, the observation that ketamine-treated mice did significantly better than controls as juveniles suggests that the effects of ketamine in adult populations may not translate to the adolescent period. Such data would suggest that there may be ways to parse the amnestic and cognitive-impairing effects of ketamine from its properties on other domains. As ketamine is emerging as a potential therapeutic agent for depression, this ability to determine which factors mediate its beneficial vs detrimental effects will be critical. For example, future studies could determine which factors in normal brain development are sensitive or conversely insensitive to ketamine-induced alterations in cognitive capacity. It is important to note that several measures indicate that the test itself was a valid assessment of spatial working memory: mice showed significant improvement in performance over sequential testing days and trials, indicating that procedural learning took place.

Finally, we investigated the hypothesis that a delayed onset of abnormal patterns of EEG would emerge as a result of chronic ketamine abuse in the juvenile period. Murine ERP data in the current study are consistent with findings in clinical studies of schizophrenia patients.^66, 67, 68, 69^ Our data provide potential insight to ketamine-related changes in brain function as manifested by theta oscillations that are thought to reflect regional connectivity. Specifically, ketamine-treated mice showed a significant failure to progress normally with age for theta coherence, as well as induced and ERSP measures. Control mice showed a reduction on these measures on reaching adulthood, but ketamine-treated mice did not. Induced activity reflects activity in the brain that is not temporally tethered to the activity of the sensory system.^70^ As such, it is thought to reflect orienting, and selectively attentional response to the occurrence of an event, rather than the sensory registration associated with that event.^70, 71^ Thus, the ketamine-treated mice express a juvenile-like pattern of ‘over reacting' to a non-threatening stimulus. A previous study showed that MK801 disrupts normal novel object recognition ability in rodents under acute exposure, and that its administration is known to cause attentional problems.^72^ Our data is not consistent with lasting attentional deficits shown in this study, but attentional changes are observed in the increased induced theta activity, indicating that attention pathways may be modified by NMDAR antagonists. Similarly, our group previously demonstrated that acute ketamine administration in adult mice yields a reduction of theta activity, indicating that the drug has a consistent influence on generators of theta activity, even though the drug's effects are different when exposure takes place in adulthood.^46^

Another ‘failure to progress' phenotype was seen in the P80 ERP measure, in that ketamine mice did not exhibit the same age-related reduction as controls. Changes in P80 component amplitude with age have been observed in human ERP studies.^73, 74^ Although the direction of change is not the same as described here in mice, the developmental time course for that change is similar. These data support the idea that although the effects of juvenile ketamine exposure may not appear in adolescence, they do emerge in adulthood. Furthermore, this P80 ERP change with age may be related to progressive deficits in the ability of subjects to withdraw attentional resources from stimuli, such as the auditory stimuli presented during EEG testing.^75^ The ketamine-related P80 changes are similar to the pattern of changes in the water maze. Ketamine-treated mice have a slightly lower P80 as juveniles compared with controls, and in adulthood both control and experimental mice show similar results. This similarity across experiments further supports the conclusion that ketamine abuse in adolescence causes a failure of age-related changes to occur in mice.

Based on cross-sectional similarities between acute manifestation of NMDAR antagonists and prodromal symptoms, previous investigators have proposed that changes in NMDAR activity may occur before symptoms appear.^76^ In our model, we restricted the NMDAR hypofunction only to the late adolescent period. In this scenario, although there were no manifestations of cognitive deficits in mice as juveniles, inappropriate hyper-social activity and a failure of attentional systems to develop normally in adulthood was observed. These data suggest that alterations in NMDAR activity that have been proposed to begin during the schizophrenia prodrome are consistent with a post-adolescent emergence of deficits. Alternatively, the lasting decline in cognition and theta power that occurs following chronic ketamine in adulthood,^42^ was not observed when chronic ketamine was restricted to the juvenile period. These data suggest that any alteration in NMDAR activity that may begin during the prodromal period in schizophrenia likely persists into adulthood. As such, the current model of adolescent ketamine abuse may not accurately reflect the chronic and persistent nature of clinical schizophrenia in humans. Similarly, the administration of NMDAR antagonists at different life stages can have drastically different effects. Data suggest that ketamine does not have the same level or kind of lasting effects on the juvenile brain as it does on the adult brain, perhaps due to increased plasticity of the juvenile brain. However, there may be a different critical period for the effects of ketamine than the period of development used in this study, which may explain the lower impact changes observed in the cognitive and social function of our subjects.

## Conclusion

The aim of our study was to evaluate the effects of juvenile ketamine abuse on social behavior, working memory and EEG during and after adolescence in mice. We hypothesized that the administration of chronic ketamine in the juvenile period would result in a delayed incidence of prominent cognitive changes in the post-pubertal period. We found several interesting changes: hypersociality in adulthood and a failure to progress to normal theta and P80 activity as reflected in EEG measures. However, our results did not show the dramatic differences we expected to observe between control and ketamine-treated mice. Our findings indicate that ketamine abuse in the juvenile period does not produce the same kind of cognitive deficits as observed in post-pubertal abuse, perhaps due to the increased plasticity of the developing brain. Interestingly, we recently found that NMDA receptor function was decreased in mice treated with ketamine in adulthood, while it was increased in those treated in juvenile period.^77^ Future studies on the effects of ketamine in the juvenile period might focus on the molecular changes in brain at different stages of development, adjust the timing of drug administration in the juvenile period or use different testing batteries that reveal effects of the drug on long-term memory and cognition.

## References

[bib1] US Department of Justice, Drug Enforcement Administration. Drugs of Abuse: A DEA Resource Guide. Drug Enforcement Administration: Springfield, VA, 2011 edn.2011.

[bib2] Archer JR, Dargan PI, Hudson S, Wood DM. Analysis of anonymous pooled urine from portable urinals in central London confirms the significant use of novel psychoactive substances. QJM 2013; 106: 147–152. 23178933 10.1093/qjmed/hcs219

[bib3] Lua AC, Lin HR, Tseng YT, Hu AR, Yeh PC. Profiles of urine samples from participants at rave party in Taiwan: prevalence of ketamine and MDMA abuse. Forensic Sci Int 2003; 136: 47–51. 12969619 10.1016/s0379-0738(03)00261-5

[bib4] World Health Organization (WHO). Ketamine: Expert Peer Review on Critical Review Report. WHO: Hammamet, Tunisia, June 4-8, 2012. 35th ECDD Agenda Item 422012..

[bib5] Tang WK, Liang HJ, Lau CG, Tang A, Ungvari GS. Relationship between cognitive impairment and depressive symptoms in current ketamine users. J Stud Alcohol Drugs 2012; 73: 460–468. 10.15288/jsad.2013.74.460 23490576

[bib6] Torjesen I. Ketamine helps a third of patients with treatment resistant depression, finds small UK study. BMJ 2014; 348: g2576. 24699320 10.1136/bmj.g2576

[bib7] Price RB, Iosifescu DV, Murrough JW, Chang LC, Al Jurdi RK, Iqbal SZ et al. Effects of ketamine on explicit and implicit suicidal cognition: a randomized controlled trial in treatment-resistant depression. Depress Anxiety 2014; 31: 335–343. 24668760 10.1002/da.22253 PMC4112410

[bib8] Jeevakumar V, Driskill C, Paine A, Sobhanian M, Vakil H, Morris B et al. Ketamine administration during the second postnatal week induces enduring schizophrenia-like behavioral symptoms and reduces parvalbumin expression in the medial prefrontal cortex of adult mice. Behav Brain Res 2015; 282: 165–175. 25591475 10.1016/j.bbr.2015.01.010

[bib9] Xu K, Krystal JH, Ning Y, Chen da C, He H, Wang D et al. Preliminary analysis of positive and negative syndrome scale in ketamine-associated psychosis in comparison with schizophrenia. J Psychiatr Res 2015; 61: 64–72. 25560772 10.1016/j.jpsychires.2014.12.012 PMC4445679

[bib10] Zugno AI, Juliao RF, Budni J, Volpato AM, Fraga DB, Pacheco FD et al. Rivastigmine reverses cognitive deficit and acetylcholinesterase activity induced by ketamine in an animal model of schizophrenia. Metab Brain Dis 2013; 28: 501–508. 23775300 10.1007/s11011-013-9417-z

[bib11] Frohlich J, Van Horn JD. Reviewing the ketamine model for schizophrenia. J Psychopharmacol 2014; 28: 287–302. 24257811 10.1177/0269881113512909 PMC4133098

[bib12] Featherstone RE, Nagy LR, Hahn CG, Siegel SJ. Juvenile exposure to ketamine causes delayed emergence of EEG abnormalities during adulthood in mice. Drug Alcohol Depend 2014; 134: 123–127. 24210161 10.1016/j.drugalcdep.2013.09.017 PMC4009692

[bib13] Featherstone RE, Liang Y, Saunders JA, Tatard-Leitman VM, Ehrlichman RS, Siegel SJ. Subchronic ketamine treatment leads to permanent changes in EEG, cognition and the astrocytic glutamate transporter EAAT2 in mice. Neurobiol Dis 2012; 47: 338–346. 22627142 10.1016/j.nbd.2012.05.003

[bib14] Jeevakumar V, Kroener S. Ketamine administration during the second postnatal week alters synaptic properties of fast-spiking interneurons in the medial prefrontal cortex of adult mice. Cereb Cortex 2014; 282: 165–175. 10.1093/cercor/bhu293 25477370

[bib15] Morgan CJ, Muetzelfeldt L, Curran HV. Ketamine use, cognition and psychological wellbeing: a comparison of frequent, infrequent and ex-users with polydrug and non-using controls. Addiction 2009; 104: 77–87. 19133891 10.1111/j.1360-0443.2008.02394.x

[bib16] Morgan CJ, Muetzelfeldt L, Curran HV. Consequences of chronic ketamine self-administration upon neurocognitive function and psychological wellbeing: a 1-year longitudinal study. Addiction 2010; 105: 121–133. 19919593 10.1111/j.1360-0443.2009.02761.x

[bib17] Venancio C, Magalhaes A, Antunes L, Summavielle T. Impaired spatial memory after ketamine administration in chronic low doses. Curr Neuropharmacol 2011; 9: 251–255. 21886600 10.2174/157015911795016912 PMC3137193

[bib18] Hiyoshi T, Hikichi H, Karasawa J, Chaki S. Metabotropic glutamate receptors regulate cortical gamma hyperactivities elicited by ketamine in rats. Neurosci Lett 2014; 567: 30–34. 24680852 10.1016/j.neulet.2014.03.025

[bib19] Morgan CJ, Duffin S, Hunt S, Monaghan L, Mason O, Curran HV. Neurocognitive function and schizophrenia-proneness in individuals dependent on ketamine, on high potency cannabis ('skunk') or on cocaine. Pharmacopsychiatry 2012; 45: 269–274. 22511328 10.1055/s-0032-1306310

[bib20] van Nimwegen L, de Haan L, van Beveren N, van den Brink W, Linszen D. [Adolescence, schizophrenia and drug abuse: interactive vulnerability. A hypothesis]. Tijdschr Psychiatr 2007; 49: 169–178. 17370223

[bib21] Squeglia LM, Jacobus J, Tapert SF. The influence of substance use on adolescent brain development. Clin EEG Neurosci 2009; 40: 31–38. 19278130 10.1177/155005940904000110 PMC2827693

[bib22] Fredriksson A, Ponten E, Gordh T, Eriksson P. Neonatal exposure to a combination of N-methyl-D-aspartate and gamma-aminobutyric acid type A receptor anesthetic agents potentiates apoptotic neurodegeneration and persistent behavioral deficits. Anesthesiology 2007; 107: 427–436. 17721245 10.1097/01.anes.0000278892.62305.9c

[bib23] Huang L, Liu Y, Jin W, Ji X, Dong Z. Ketamine potentiates hippocampal neurodegeneration and persistent learning and memory impairment through the PKCgamma-ERK signaling pathway in the developing brain. Brain Res 2012; 1476: 164–171. 22985497 10.1016/j.brainres.2012.07.059

[bib24] Paule MG, Li M, Allen RR, Liu F, Zou X, Hotchkiss C et al. Ketamine anesthesia during the first week of life can cause long-lasting cognitive deficits in rhesus monkeys. Neurotoxicol Teratol 2011; 33: 220–230. 21241795 10.1016/j.ntt.2011.01.001 PMC3071878

[bib25] Young C, Jevtovic-Todorovic V, Qin YQ, Tenkova T, Wang H, Labruyere J et al. Potential of ketamine and midazolam, individually or in combination, to induce apoptotic neurodegeneration in the infant mouse brain. Br J Pharmacol 2005; 146: 189–197. 15997239 10.1038/sj.bjp.0706301 PMC1576258

[bib26] Scallet AC, Schmued LC, Slikker W Jr, Grunberg N, Faustino PJ, Davis H et al. Developmental neurotoxicity of ketamine: morphometric confirmation, exposure parameters, and multiple fluorescent labeling of apoptotic neurons. Toxicol Sci 2004; 81: 364–370. 15254342 10.1093/toxsci/kfh224

[bib27] Zou X, Patterson TA, Sadovova N, Twaddle NC, Doerge DR, Zhang X et al. Potential neurotoxicity of ketamine in the developing rat brain. Toxicol Sci 2009; 108: 149–158. 19126600 10.1093/toxsci/kfn270 PMC2721655

[bib28] Jin J, Gong K, Zou X, Wang R, Lin Q, Chen J. The blockade of NMDA receptor ion channels by ketamine is enhanced in developing rat cortical neurons. Neurosci Lett 2013; 539: 11–15. 23395831 10.1016/j.neulet.2013.01.034 PMC3602117

[bib29] Green SM, Rothrock SG, Harris T, Hopkins GA, Garrett W, Sherwin T. Intravenous ketamine for pediatric sedation in the emergency department: safety profile with 156 cases. Acad Emerg Med 1998; 5: 971–976. 9862587 10.1111/j.1553-2712.1998.tb02773.x

[bib30] Green SM, Rothrock SG, Lynch EL, Ho M, Harris T, Hestdalen R et al. Intramuscular ketamine for pediatric sedation in the emergency department: safety profile in 1,022 cases. Ann Emerg Med 1998; 31: 688–697. 9624307 10.1016/s0196-0644(98)70226-4

[bib31] Green SM, Krauss B. Clinical practice guideline for emergency department ketamine dissociative sedation in children. Ann Emerg Med 2004; 44: 460–471. 15520705 10.1016/S0196064404006365

[bib32] Craven R. Ketamine. Anaesthesia 2007; 62: 48–53. 17937714 10.1111/j.1365-2044.2007.05298.x

[bib33] Petrack EM, Marx CM, Wright MS. Intramuscular ketamine is superior to meperidine, promethazine, and chlorpromazine for pediatric emergency department sedation. Arch Pediatr Adolesc Med 1996; 150: 676–681. 8673189 10.1001/archpedi.1996.02170320022003

[bib34] Chen GH, Wang YJ, Wang XM, Zhou JN. Accelerated senescence prone mouse-8 shows early onset of deficits in spatial learning and memory in the radial six-arm water maze. Physiol Behav 2004; 82: 883–890. 15451654 10.1016/j.physbeh.2004.07.008

[bib35] Shukitt-Hale B, McEwen JJ, Szprengiel A, Joseph JA. Effect of age on the radial arm water maze-a test of spatial learning and memory. Neurobiol Aging 2004; 25: 223–229. 14749140 10.1016/s0197-4580(03)00041-1

[bib36] Kim S, Pickup S, Fairless AH, Ittyerah R, Dow HC, Abel T et al. Association between sociability and diffusion tensor imaging in BALB/cJ mice. NMR Biomed 2012; 25: 104–112. 21618305 10.1002/nbm.1722 PMC4188421

[bib37] Sankoorikal GM, Kaercher KA, Boon CJ, Lee JK, Brodkin ES. A mouse model system for genetic analysis of sociability: C57BL/6J versus BALB/cJ inbred mouse strains. Biol Psychiatry 2006; 59: 415–423. 16199013 10.1016/j.biopsych.2005.07.026

[bib38] Fairless AH, Shah RY, Guthrie AJ, Li H, Brodkin ES. Deconstructing sociability, an autism-relevant phenotype, in mouse models. Anat Rec (Hoboken) 2011; 294: 1713–1725. 21905241 10.1002/ar.21318 PMC3176979

[bib39] Uhlhaas PJ, Singer W. The development of neural synchrony and large-scale cortical networks during adolescence: relevance for the pathophysiology of schizophrenia and neurodevelopmental hypothesis. Schizophr Bull 2011; 37: 514–523. 21505118 10.1093/schbul/sbr034 PMC3080681

[bib40] Uhlhaas PJ, Roux F, Rodriguez E, Rotarska-Jagiela A, Singer W. Neural synchrony and the development of cortical networks. Trends Cogn Sci 2010; 14: 72–80. 20080054 10.1016/j.tics.2009.12.002

[bib41] Uhlhaas PJ, Singer W. Neural synchrony in brain disorders: relevance for cognitive dysfunctions and pathophysiology. Neuron 2006; 52: 155–168. 17015233 10.1016/j.neuron.2006.09.020

[bib42] Lazarewicz MT, Ehrlichman RS, Maxwell CR, Gandal MJ, Finkel LH, Siegel SJ. Ketamine modulates theta and gamma oscillations. J Cogn Neurosci 2010; 22: 1452–1464. 19583475 10.1162/jocn.2009.21305

[bib43] Ehrlichman RS, Gandal MJ, Maxwell CR, Lazarewicz MT, Finkel LH, Contreras D et al. N-methyl-d-aspartic acid receptor antagonist-induced frequency oscillations in mice recreate pattern of electrophysiological deficits in schizophrenia. Neuroscience 2009; 158: 705–712. 19015010 10.1016/j.neuroscience.2008.10.031

[bib44] Featherstone RE, MT-L V, Suh JD, Lin R, Lucki I, Siegel SJ. Electrophysiological and behavioral responses to ketamine in mice with reduced Akt1 expression. Psychopharmacology (Berl) 2013; 227: 639–649. 23392353 10.1007/s00213-013-2997-9 PMC3808977

[bib45] Saunders JA, Gandal MJ, Roberts TP, Siegel SJ. NMDA antagonist MK801 recreates auditory electrophysiology disruption present in autism and other neurodevelopmental disorders. Behav Brain Res 2012; 234: 233–237. 22771812 10.1016/j.bbr.2012.06.032 PMC4124897

[bib46] Saunders JA, Gandal MJ, Siegel SJ. NMDA antagonists recreate signal-to-noise ratio and timing perturbations present in schizophrenia. Neurobiol Dis 2012; 46: 93–100. 22245663 10.1016/j.nbd.2011.12.049 PMC4161042

[bib47] Tesche CD, Karhu J. Theta oscillations index human hippocampal activation during a working memory task. Proc Natl Acad Sci USA 2000; 97: 919–924. 10639180 10.1073/pnas.97.2.919 PMC15431

[bib48] Poch C, Fuentemilla L, Barnes GR, Duzel E. Hippocampal theta-phase modulation of replay correlates with configural-relational short-term memory performance. J Neurosci 2011; 31: 7038–7042. 21562265 10.1523/JNEUROSCI.6305-10.2011 PMC6703212

[bib49] Fuentemilla L, Penny WD, Cashdollar N, Bunzeck N, Duzel E. Theta-coupled periodic replay in working memory. Curr Biol 2010; 20: 606–612. 20303266 10.1016/j.cub.2010.01.057 PMC2856918

[bib50] Duzel E, Penny WD, Burgess N. Brain oscillations and memory. Curr Opin Neurobiol 2010; 20: 143–149. 20181475 10.1016/j.conb.2010.01.004

[bib51] Guderian S, Duzel E. Induced theta oscillations mediate large-scale synchrony with mediotemporal areas during recollection in humans. Hippocampus 2005; 15: 901–912. 16161060 10.1002/hipo.20125

[bib52] Monte AS, de Souza GC, McIntyre RS, Soczynska JK, dos Santos JV, Cordeiro RC et al. Prevention and reversal of ketamine-induced schizophrenia related behavior by minocycline in mice: Possible involvement of antioxidant and nitrergic pathways. J Psychopharmacol 2013; 27: 1032–1043. 24045882 10.1177/0269881113503506

[bib53] Bian SZ, Liu WL, Zhang ZX, Gu ZL, Jiang XG, Guo CY. [The correlation between ketamine-induced schizophrenia-like signs in mice and the expressions of NRG1, ErbB4 mRNA]. Fa Yi Xue Za Zhi. Fa Yi Xue Za Zhi 2009; 25: 348–351, 358. 20000043

[bib54] Gandal MJ, Edgar JC, Klook K, Siegel SJ. Gamma synchrony: towards a translational biomarker for the treatment-resistant symptoms of schizophrenia. Neuropharmacology 2012; 62: 1504–1518. 21349276 10.1016/j.neuropharm.2011.02.007 PMC3264822

[bib55] Jeffrey M, Lang M, Gane J, Wu C, Burnham WM, Zhang L. A reliable method for intracranial electrode implantation and chronic electrical stimulation in the mouse brain. BMC Neurosci 2013; 14: 82. 23914984 10.1186/1471-2202-14-82 PMC3750568

[bib56] Wu C, Wais M, Sheppy E, del Campo M, Zhang L. A glue-based, screw-free method for implantation of intra-cranial electrodes in young mice. J Neurosci Methods 2008; 171: 126–131. 18420280 10.1016/j.jneumeth.2008.03.001

[bib57] Roach BJ, Mathalon DH. Event-related EEG time-frequency analysis: an overview of measures and an analysis of early gamma band phase locking in schizophrenia. Schizophr Bull 2008; 34: 907–926. 18684772 10.1093/schbul/sbn093 PMC2632478

[bib58] Brodkin ES, Hagemann A, Nemetski SM, Silver LM. So cial approach-avoidance behavior of inbred mouse strains towards DBA/2 mice. Brain Res 2004; 1002: 151–157. 14988045 10.1016/j.brainres.2003.12.013

[bib59] Semple BD, Blomgren K, Gimlin K, Ferriero DM, Noble-Haeusslein LJ. Brain development in rodents and humans: Identifying benchmarks of maturation and vulnerability to injury across species. Prog Neurobiol 2013; 106-107: 1–16. 23583307 10.1016/j.pneurobio.2013.04.001 PMC3737272

[bib60] Ebert A, Haussleiter IS, Juckel G, Brune M, Roser P. Impaired facial emotion recognition in a ketamine model of psychosis. Psychiatry Res 2012; 200: 724–727. 22776754 10.1016/j.psychres.2012.06.034

[bib61] Moy SS, Nonneman RJ, Shafer GO, Nikolova VD, Riddick NV, Agster KL et al. Disruption of social approach by MK-801, amphetamine, and fluoxetine in adolescent C57BL/6J mice. Neurotoxicol Teratol 2013; 36: 36–46. 22898204 10.1016/j.ntt.2012.07.007 PMC3509253

[bib62] Kohler CG, Bilker W, Hagendoorn M, Gur RE, Gur RC. Emotion recognition deficit in schizophrenia: association with symptomatology and cognition. Biol Psychiatry 2000; 48: 127–136. 10903409 10.1016/s0006-3223(00)00847-7

[bib63] Sachs G, Steger-Wuchse D, Kryspin-Exner I, Gur RC, Katschnig H. Facial recognition deficits and cognition in schizophrenia. Schizophr Res 2004; 68: 27–35. 15037337 10.1016/S0920-9964(03)00131-2

[bib64] Gresack JE, Frick KM. Male mice exhibit better spatial working and reference memory than females in a water-escape radial arm maze task. Brain Res 2003; 982: 98–107. 12915244 10.1016/s0006-8993(03)03000-2

[bib65] Fine JM, Baillargeon AM, Renner DB, Hoerster NS, Tokarev J, Colton S et al. Intranasal deferoxamine improves performance in radial arm water maze, stabilizes HIF-1alpha, and phosphorylates GSK3beta in P301L tau transgenic mice. Exp Brain Res 2012; 219: 381–390. 22547371 10.1007/s00221-012-3101-0

[bib66] Kiang M, Braff DL, Sprock J, Light GA. The relationship between preattentive sensory processing deficits and age in schizophrenia patients. Clin Neurophysiol 2009; 120: 1949–1957. 19786365 10.1016/j.clinph.2009.08.019 PMC2902236

[bib67] Turetsky BI, Dress EM, Braff DL, Calkins ME, Green MF, Greenwood TA et al. The utility of P300 as a schizophrenia endophenotype and predictive biomarker: Clinical and socio-demographic modulators in COGS-2. Schizophr Res 2015; 163: 53–62. 25306203 10.1016/j.schres.2014.09.024 PMC4382423

[bib68] Light GA, Swerdlow NR. Future clinical uses of neurophysiological biomarkers to predict and monitor treatment response for schizophrenia. Ann NY Acad Sci 2015; 1344: 105–119. 25752648 10.1111/nyas.12730 PMC4412775

[bib69] Light GA, Swerdlow NR, Thomas ML, Calkins ME, Green MF, Greenwood TA et al. Validation of mismatch negativity and P3a for use in multi-site studies of schizophrenia: characterization of demographic, clinical, cognitive, and functional correlates in COGS-2. Schizophr Res 2015; 163: 63–72. 25449710 10.1016/j.schres.2014.09.042 PMC4382452

[bib70] Basar E, Basar-Eroglu C, Karakas S, Schurmann M. Gamma, alpha, delta, and theta oscillations govern cognitive processes. Int J Psychophysiol 2001; 39: 241–248. 11163901 10.1016/s0167-8760(00)00145-8

[bib71] Kemp IR, Kaada BR. The relation of hippocampal theta activity to arousal, attentive behaviour and somato-motor movements in unrestrained cats. Brain Res 1975; 95: 323–342. 168940 10.1016/0006-8993(75)90110-9

[bib72] Neill JC, Barnes S, Cook S, Grayson B, Idris NF, McLean SL et al. Animal models of cognitive dysfunction and negative symptoms of schizophrenia: focus on NMDA receptor antagonism. Pharmacol Ther 2010; 128: 419–432. 20705091 10.1016/j.pharmthera.2010.07.004

[bib73] Anderer P, Semlitsch HV, Saletu B. Multichannel auditory event-related brain potentials: effects of normal aging on the scalp distribution of N1, P2, N2 and P300 latencies and amplitudes. Electroencephalogr Clin Neurophysiol 1996; 99: 458–472. 9020805 10.1016/s0013-4694(96)96518-9

[bib74] Crowley KE, Colrain IM. A review of the evidence for P2 being an independent component process: age, sleep and modality. Clin Neurophysiol 2004; 115: 732–744. 15003751 10.1016/j.clinph.2003.11.021

[bib75] Garcia-Larrea L, Lukaszewicz AC, Mauguiere F. Revisiting the oddball paradigm. Non-target vs neutral stimuli and the evaluation of ERP attentional effects. Neuropsychologia 1992; 30: 723–741. 1407488 10.1016/0028-3932(92)90042-k

[bib76] Bodatsch M, Klosterkotter J, Daumann J. Contributions of experimental psychiatry to research on the psychosis prodrome. Front Psychiatry 2013; 4: 170. 24381564 10.3389/fpsyt.2013.00170 PMC3865446

[bib77] Banerjee A, Wang HY, Borgmann-Winter KE, MacDonald ML, Kaprielian H, Stucky A et al. Src kinase as a mediator of convergent molecular abnormalities leading to NMDAR hypoactivity in schizophrenia. Mol Psychiatry 2014; 20: 1091–1100. 25330739 10.1038/mp.2014.115 PMC5156326

