# Supplementary figures and images for: Delayed emergence of behavioral and electrophysiological effects following juvenile ketamine exposure in mice
Source: Transl Psychiatry. 2015 Sep 15;5(9):e635–. doi: 10.1038/tp.2015.111 (PMC5068812; doi:10.1038/tp.2015.111)

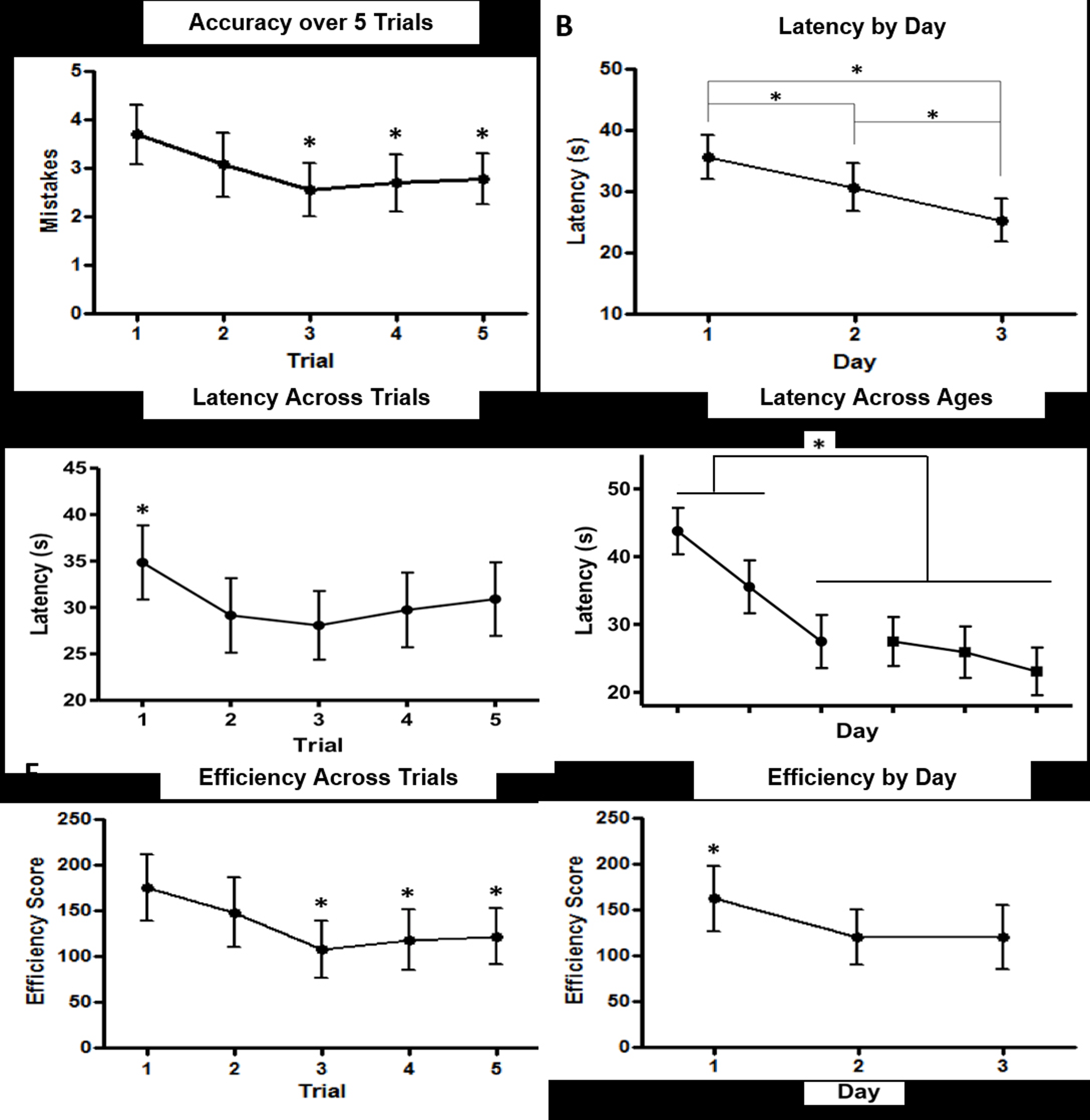

Supplement: Supplementary Figure 1 [file tp2015111x1.tif]
