# Supplementary material for: Delayed emergence of behavioral and electrophysiological effects following juvenile ketamine exposure in mice
Source: Transl Psychiatry. 2015 Sep 15;5(9):e635–. doi: 10.1038/tp.2015.111 (PMC5068812; doi:10.1038/tp.2015.111)
Supplement: Supplementary Information [file tp2015111x2.doc]

**Supplemental Figure 1.** Additional Six Arm Radial Water Maze Data. Ketamine-treated mice are represented in white, and controls in black. **Figure 1A:** Accuracy across 5 trials, averaging trials for 3 days of testing. Number of mistakes significantly decreased in later trials across both ages, and all days (F_4,92_=5.2035, p=0.0008, posthoc: p<0.002 comparing trials 1-2 to trials 3-5, n.s. between trials 1-2 and trials 3-5). **Figure 1B:** Latency in completion of the water maze task with averaged trials on both sets of 3 testing days. Latency in completing the water maze task decreased significantly each day of both testing periods, across both treatment groups (F_2,46_=24.254, p=0.00000, posthoc: p<0.05 for day 1 vs 2 or 3 and day 2 vs 3). Time spent completing the maze decreased steadily during the 3 testing days, across group. **Figure 1C:** Latency to maze completion across 5 trials, averaged over both 3 day testing periods. Time spent completing the maze decreased significantly after the first trial on each day of the testing period (F_4,92_=3.9955, p=0.00493). This effect was observed across both ages and treatment groups. **Figure 1D**: Latency in juvenile mice (circle) and adult mice (square), averaged across both treatment groups. The average latency of all mice during an entire day of testing was observed to significantly decrease by day 3 of the juvenile testing period, and remain at this optimal level throughout 3 days of adulthood testing (F_2,46_=.4534, p=0.01706). **Figure 1E:** Efficiency scores across trials, averaged over both 3 day testing periods. All mice showed a significant increase in efficiency, indicated by a lower efficiency score by trial 3 each day without further improvement (F_4,92_=4.1047, p=0.00418, posthoc: p<0.05 for trial 1 and 2 vs trials 3-5, n.s. between 1 and 2 or 3-5). **Figure 1F:** Efficiency scores compared across 3 days of testing. All mice show a significant improvement in efficiency on days 2 and 3 of testing, as compared to day 1, across both juvenile and adulthood (F_2,46_=4.1934, p=0.02124, posthoc: p<0.05, day 1 vs 2 or 3).
